# Isolation and culture of pure adult mouse microglia and astrocytes for *in vitro* characterization and analyses

**DOI:** 10.1016/j.xpro.2022.101295

**Published:** 2022-04-11

**Authors:** Mark T. Milner, Grace MEP. Lawrence, Caroline L. Holley, Liviu-Gabriel Bodea, Jürgen Götz, Sabrina S. Burgener, Kate Schroder

**Affiliations:** 1 Institute for Molecular Bioscience and IMB Centre for Inflammation and Disease Research, The University of Queensland, St. Lucia, QLD 4072, Australia; 2 Clem Jones Centre for Ageing and Dementia Research, Queensland Brain Institute, The University of Queensland, St. Lucia, QLD 4072, Australia

**Keywords:** Cell Biology, Cell culture, Cell isolation, Single Cell, Flow Cytometry/Mass Cytometry, Immunology, Neuroscience

## Abstract

Microglia and astrocytes are implicated in aging and age-related diseases. Here, we present a protocol to isolate and culture these glia cells from the murine brain. The protocol consists of two parts: magnetic sorting of adult microglia and mechanical/magnetic sorting of adult microglia and astrocytes. We then describe the characterization of these glial cells by flow cytometry and immunohistochemistry. Microglia isolated from aged mice maintain age-related phenotype during culture. These purified glia cells can be applied in *ex vivo* studies.

## Highlights

• Simple protocol to isolate and culture adult and aged murine microglia and astrocytes
• Microglia maintain their age-related phenotype after culture period
• Aged microglia and astrocytes co-purified from the same mouse can be studied *ex vivo*

## Graphical abstract

**Figure:**
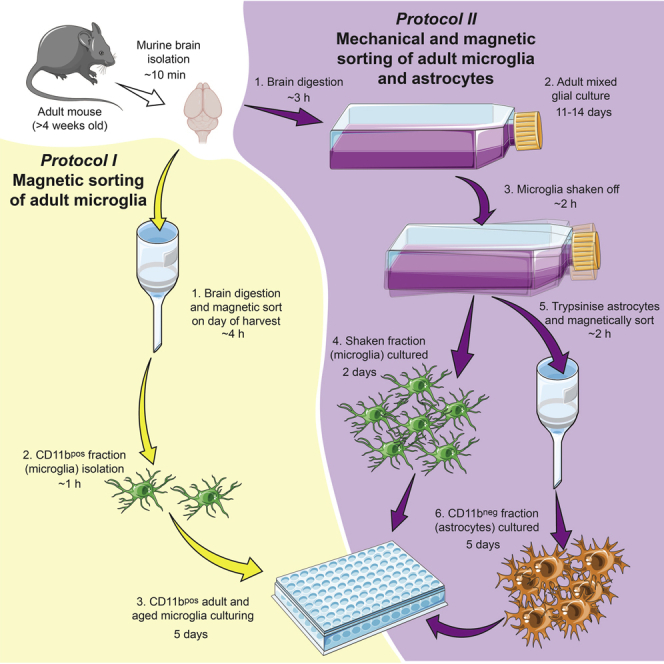

## Before you begin

Ensure you have received the appropriate institutional permissions to work with animal tissues before proceeding with this protocol.

Prepare the Adult Glial Media (AGM) and MACS buffer as described below under Materials and equipment and cool the centrifuge to 4°C prior to starting the experiment.

### Institutional permissions (if applicable)

The University of Queensland’s Animal Ethics Committee approved all the experimental protocols described below (2019/AE000281).

## Key resources table

**Table:** 

REAGENT or RESOURCE	SOURCE	IDENTIFIER
**Antibodies**		

CD45 FITC (1:800)	BioLegend	Clone 30-F11, Cat.#103108; RRID: AB_312973
CD49a PerCP-Cy5.5 (1:400)	BioLegend	Clone HMa1, Cat.#142611; RRID: AB_2750219
CD11b BV421 (1:800)	BioLegend	Clone: M1/70, Cat.#101236; RRID: AB_11203704
CD45 BV510 (1:800)	BioLegend	Clone: 30-F11, Cat.# 103138; RRID: AB_2563061
CD68 BV605 (1:400)	BioLegend	Clone: FA-11, Cat.#137021; RRID: AB_2616811
CD11b APC (1:800)	BioLegend	Clone: M1/70, Cat.#101212; RRID: AB_312795
I-A/I-E (MHCII) APC (1:400)	BioLegend	Clone:M5/114.15.2, Cat.#107614; RRID: AB_313329
O4 APC (1:75)	Miltenyi Biotec	Clone: O4, Cat.#130-119-155; RRID: AB_2751644
FVS700 (1:1000)	BD Bioscience (Becton, Dickinson & Company)	Cat.#564997; RRID: AB_2869637
GLAST PE (1:75)	Miltenyi Biotec	Clone: ACSA-1, Cat.#130-118-344; RRID: AB_2733723
CD16/32 (clone 2.4G2) (1:100)	BD Bioscience (Becton Dickinson & Company)	Clone: 2.4G2, Cat.#553142; RRID: AB_394657
Iba1 (1:500)	Wako	Cat.#019-19741; RRID: AB_839504
GFAP (1:500)	Abcam	Cat.#AB53554-100UG; RRID: AB_880202
DAPI (4′,6-diamidino-2-phenylindole) (1:1000)	Sigma-Aldrich	Cat.#D9542-5MG
Alexa Fluor 647 Phalloidin (1:40)	Thermo Fisher Scientific	Cat.#A22287; RRID: AB_2620155
Goat anti-Rabbit IgG (H+L) Cross-Adsorbed Secondary Antibody, Alexa Fluor Plus 594 (1:500)	Thermo Fisher Scientific	Cat.#A32740; RRID: AB_2762824
Chicken anti-Goat IgG (H+L) Cross-Adsorbed Secondary Antibody, Alexa Fluor 488 (1:500)	Thermo Fisher Scientific	Cat.#A-21467; RRID: AB_2535870

**Biological samples**		

C57BL/6J mouse brains (>4 weeks old), males and/or females	Mice bred in-house	N/A

**Chemicals, peptides, and recombinant proteins**		

Macrophage-colony stimulating factor (M-CSF), endotoxin-free	Prepared in-house (alternative commercial supplier: PeproTech)	N/A (PeproTech, Cat.# 315-02)
Granulocyte-macrophage-colony stimulating factor (GM-CSF)	PeproTech	Cat.#315-03
PBS-g (containing Ca^2+^, Mg^2+^, glucose, and pyruvate)	Thermo Fisher Scientific	Cat.#14287080
PBS (Ca^2+^Mg^2+^ free)	Gibco	Cat.#10010023
EDTA (0.5 M)	Gibco	Cat.#15575020
Trypan blue	Sigma-Aldrich	Cat.#T8154-100ML
DMEM	Thermo Fisher Scientific	Cat.#11995073
Fetal calf serum (FCS), endotoxin-free	Corning	Cat.#35-076-CV
Penicillin/streptomycin	Gibco	Cat.#15140122
GlutaMAX	Gibco	Cat.#35050061
0.25% Trypsin-EDTA	Thermo Fisher Scientific	Cat.#25200072
Bovine serum albumin (BSA) fraction V (7.5%)	Thermo Fisher Scientific	Cat.#15260037
FixPerm buffers	BioLegend	Cat.#426803
Poly-L-lysine solution	Sigma-Aldrich	Cat.#P4832
Pierce^TM^ 16% Formaldehyde (w/v), Methanol-free	Thermo Fisher Scientific	Cat.#28908
ProLong^TM^ Gold Antifade Mountant	Thermo Fisher Scientific	Cat.#P36934

**Critical commercial assays**		

Adult Brain Dissociation Kit	Miltenyi Biotec	Cat.#130-107-677
CD11b magnetic beads	Miltenyi Biotec	Cat.#130-049-601

**Experimental models: Organisms/strains**		

C57BL/6J breeders	The Jackson Laboratory	Cat.#000664

**Software and algorithms**		

FlowJo	BD (Becton, Dickinson & Company)	https://www.flowjo.com/
BD FACSDiva™ Software	BD (Becton, Dickinson & Company)	https://www.bdbiosciences.com/en-au/products/software/instrument-software/bd-facsdiva-software#Overview
GraphPad Prism	GraphPad	https://www.graphpad.com/scientific-software/prism/
Fiji ImageJ	(Schindelin et al., 2012) ‘Fiji: an open-source platform for biological-image analysis.’ Nature Methods, 9(7), 676–682.	https://imagej.net/software/fiji/

**Other**		

BD LSRFortessa™ X-20 Cell Analyzer	BD (Becton, Dickinson & Company)	https://www.bdbiosciences.com/en-au/products/instruments/flow-cytometers/research-cell-analyzers/bd-lsrfortessa-x-20
gentleMACS™ Octo Dissociator with Heaters	Miltenyi Biotec	https://www.miltenyibiotec.com/AU-en/products/gentlemacs-octo-dissociator-with-heaters.html#gref
MS columns	Miltenyi Biotec	Cat.#130-042-201
LS columns	Miltenyi Biotec	Cat.#130-042-401
OctoMACS Separator	Miltenyi Biotec	Cat.#130-042-109
C-tubes	Miltenyi Biotec	Cat.#130-093-237
T25 cell culture grade flasks	Thermo Fisher Scientific	Cat.#156340
70 μm cell strainers	Bio-Strategy	Cat.#BDAA352350
FACS tubes (5 mL round bottom polystyrene test tube)	In Vitro Technologies	Cat.#352052
Cell scraper	Sarstedt	Cat.#83.3951
12 mm coverslips	Bio-Strategy	Cat.#EPBRCSC121GP
Microscope slides	Bio-Strategy	Cat.#EPBRSF21201
24-well tissue culture plates	Sigma-Aldrich	Cat.#3526
Biosafety cabinet	Gelaire	Cat.#BHEN2004D

## Materials and equipment

Adult Glial Media (AGM) (store at 4°C for up to 1 month).

***Note:*** Add GM-CSF (50 ng/mL) and M-CSF (100 ng/mL) fresh to AGM immediately before use. Do not prepare AGM supplemented with GM-CSF and M-CSF for long-term storage.

**Table:** 

Reagent	Final concentration	Amount
DMEM	1×	500 mL
Fetal calf serum (FCS)	10%	50 mL
100× pen/strep	1×	5 mL
100× GlutaMAX	1×	5 mL
**Total**	**n/a**	**560 mL**

**Table:** MACS Buffer (store at 4°C for up to 6 months)

Reagent	Final concentration	Amount
PBS	1×	500 mL
Bovine Serum Albumin (BSA) fraction V (7.5%)	0.5%	33.33 mL
EDTA	2 mM	2 mL
**Total**	**n/a**	**535 mL**

**Table:** Flow cytometry antibody panel

Antibodies	Dilution factor	Volume (μL) per sample (50 μL)
CD45 FITC	1:800	0.0625
CD49a PerCPCy5.5	1:400	0.125
CD11b BV421	1:800	0.0625
CD68 BV605	1:400	0.125
CD90.2 BV780	1:800	0.0625
O4 APC	1:75	0.66
MHCII APC	1:400	0.125
FVS700 APC700	1:1,000	0.05
GLAST PE	1:75	0.66

**Table:** Immunocytochemistry antibody panel

Antibodies	Dilution factor	Final concentration
Iba1	1:500	1 μg/mL
GFAP	1:500	1 μg/mL
DAPI (4′,6-diamidino-2-phenylindole)	1:1,000	20 ng/mL
AlexaFluor^TM^647 Phalloidin	1:40	7.5 U/mL
Goat anti-Rabbit IgG (H+L) Cross-Adsorbed Secondary Antibody, Alexa FluorPlus 594	1:500	4 μg/mL
Chicken anti-Goat IgG (H+L) Cross-Adsorbed Secondary Antibody, Alexa Fluor 488	1:500	4 μg/mL

## Step-by-step method details

This method first prepares a single-cell suspension of total brain cells, using the Miltenyi Biotec Adult Brain Dissociation Kit (kits purchased from 2019 onwards). The cell suspension is then further processed to purify adult microglia and astrocytes.

### Isolation of total adult brain cells

**Timing: 4 h**

This protocol enables the successful dissociation of murine brains into single-cell suspensions. The protocol below can be used for adult (4 weeks or older) and aged mice.

**Figure 1:**
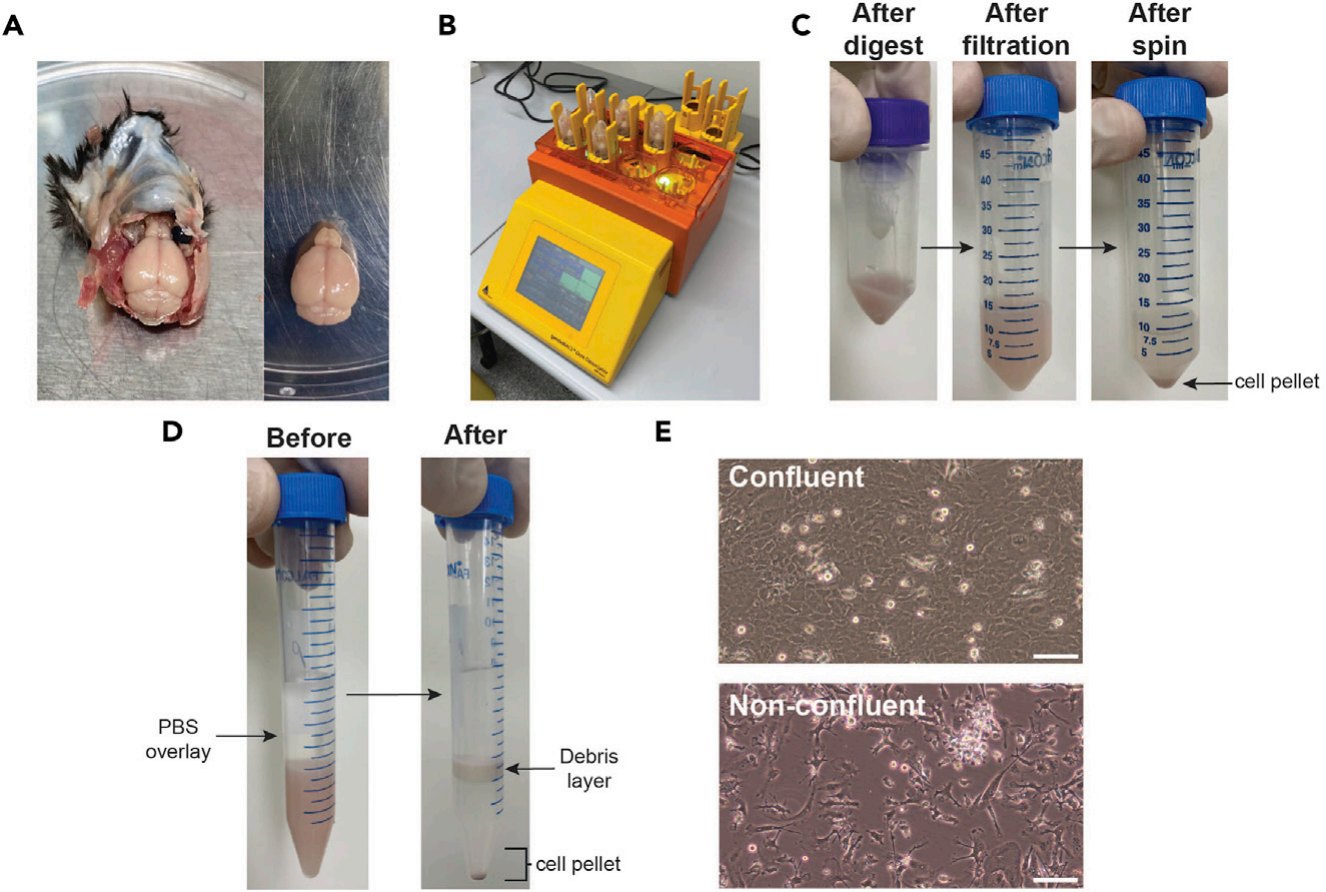
Adult glial isolation protocol (A) Adult murine brain is removed from the skull. (B) Miltenyi Biotec OctoDissociator with heaters. (C) Adult murine brain after homogenization with OctoDissociator. (D) Adult murine brain debris removal step, before and after centrifugation. (E) Examples of confluent and non-confluent mixed glial cultures after 12 days in culture. Scale bars, 50 μm.
  c. Once the brain is isolated, perform all future protocol steps within a biosafety cabinet.

1. Isolate the brain from an adult or aged mouse.
  a. Sacrifice the mouse using CO_2_. Spray the mouse with ethanol, and then decapitate it using sterile scissors.
    ***Note:*** Transcardial perfusion is not required for these protocols. Any circulating immune cells will not survive the culturing conditions and so will not affect the purity of the obtained cells (Swamydas et al., 2015; Grosjean et al., 2021; Ramezani-Rad and Rickert, 2021).
  b. Using fresh sterile scissors and forceps, remove the skull from the head (Figure 1A).

2. Obtain a bulk single-cell suspension from the brain, as per the following detailed manufacturer’s instructions for the Miltenyi Biotec Adult Brain Dissociation Kit (Figures 1B–1D):
  **CRITICAL:** All steps of the Miltenyi Biotec Adult Brain Dissociation protocol must be completed using ice-cold buffers and samples kept on ice.
  a. Place the brain in a 50 mL Falcon tube containing 1 mL ice-cold PBS-g.
  b. Prepare the enzyme mix by adding 50 μL Enzyme P, 1,900 μL Buffer Z, 20 μL Buffer Y and 10 μL Enzyme A to a C-tube.
  c. Add ×1 adult mouse brain to the C-tube and roughly chop with a scalpel.
  d. Place the C-tube on the gentleMACS Octo Dissociator machine with heaters and select program 37°C_ABDK_01 (Figure 1B).
  e. Once the program is complete (after 30 min), remove the C-tube and centrifuge it at 500 × *g* for 5 s to collect all homogenized brain contents at the bottom of the tube (Figure 1C).
  f. Resuspend the brain homogenate and filter through a 70 μm cell strainer into a 50 mL Falcon tube.
  g. Wash the C-tube with 10 mL ice-cold PBS-g and pass it through the cell strainer into the Falcon tube to collect any residual brain homogenate (Figure 1C).
  h. Centrifuge the filtered brain homogenate at 300 × *g* for 10 min at 4°C.
  i. After centrifugation, gently remove the supernatant from the brain homogenate pellet and resuspend in 1 mL ice-cold PBS-g (Figure 1C).
  j. Transfer the brain homogenate to a 15 mL Falcon tube.
  k. Top up the brain homogenate to a final volume of 3,100 μL with PBS-g.
    **CRITICAL:** To ensure efficient debris removal and maximal sample purity using the Miltenyi Biotec Adult Brain Dissociation Kit protocol, resuspend the brain tissue pellet with PBS-g to generate a total volume of 3,100 μL before adding the Debris Removal Solution. To achieve this exact volume, it is recommended to add 1 mL of PBS-g to the brain tissue pellet, resuspend the pellet thoroughly and transfer it to the 15 mL Falcon tube being used in the next step. While transferring the resuspended pellet, take note of the actual volume being transferred (usually around 1,800 μL). From there, add the appropriate amount of additional PBS to the Falcon tube to achieve a total volume of 3,100 μL.
  l. Add 900 μL Debris Removal Solution to the brain homogenate and mix well but gently.
  m. Gently overlay the brain homogenate with 4 mL PBS-g (Figure 1D).
  n. Once overlaid, centrifuge the overlaid brain homogenate at 3,000 × *g* for 10 min at 4°C with no brake.
    **CRITICAL:** During the debris removal step, cells must be centrifuged at 3,000 × *g* for 10 min at 4°C with NO BRAKE, to maximize clear cell separation (Figure 1D). Leaving the brake on disrupts the layers and makes debris removal more difficult.
  o. After centrifugation, remove the top two layers containing the debris layer and buffer (Figure 1D).
  p. Fill the 15 mL Falcon tube with PBS-g and centrifuge at 1,000 × *g* for 10 min at 4°C with brake.
  q. Remove the supernatant from the Falcon tube and resuspend the cell pellet in 1 mL 1× Red Blood Cell Removal solution.
  r. Incubate at 4°C for 10 min.
  s. After incubation, add 10 mL PBS containing 1% FCS to stop the red blood cell lysis, and centrifuge the Falcon tube at 300 × *g* for 10 min at 4°C.
  t. The cell pellet now contains the total single-cell suspension from the adult brain.
3. Either directly isolate microglia using a CD11b magnetic sort (**Protocol I**), or culture total brain cells in flasks as an adult mixed glial culture from which microglia and astrocytes are purified at day 12 (**Protocol II**).

### Protocol I: Isolation of adult microglia using magnetic sorting

**Timing: 5 days**

This protocol enables the successful culture and isolation of pure adult microglia for *in vitro* characterization using a magnetic sort system to positively select microglia that are CD11b^pos^ (https://www.miltenyibiotec.com/AU-en/products/cd11b-microbeads-human-and-mouse.html#gref).

**Figure 2:**
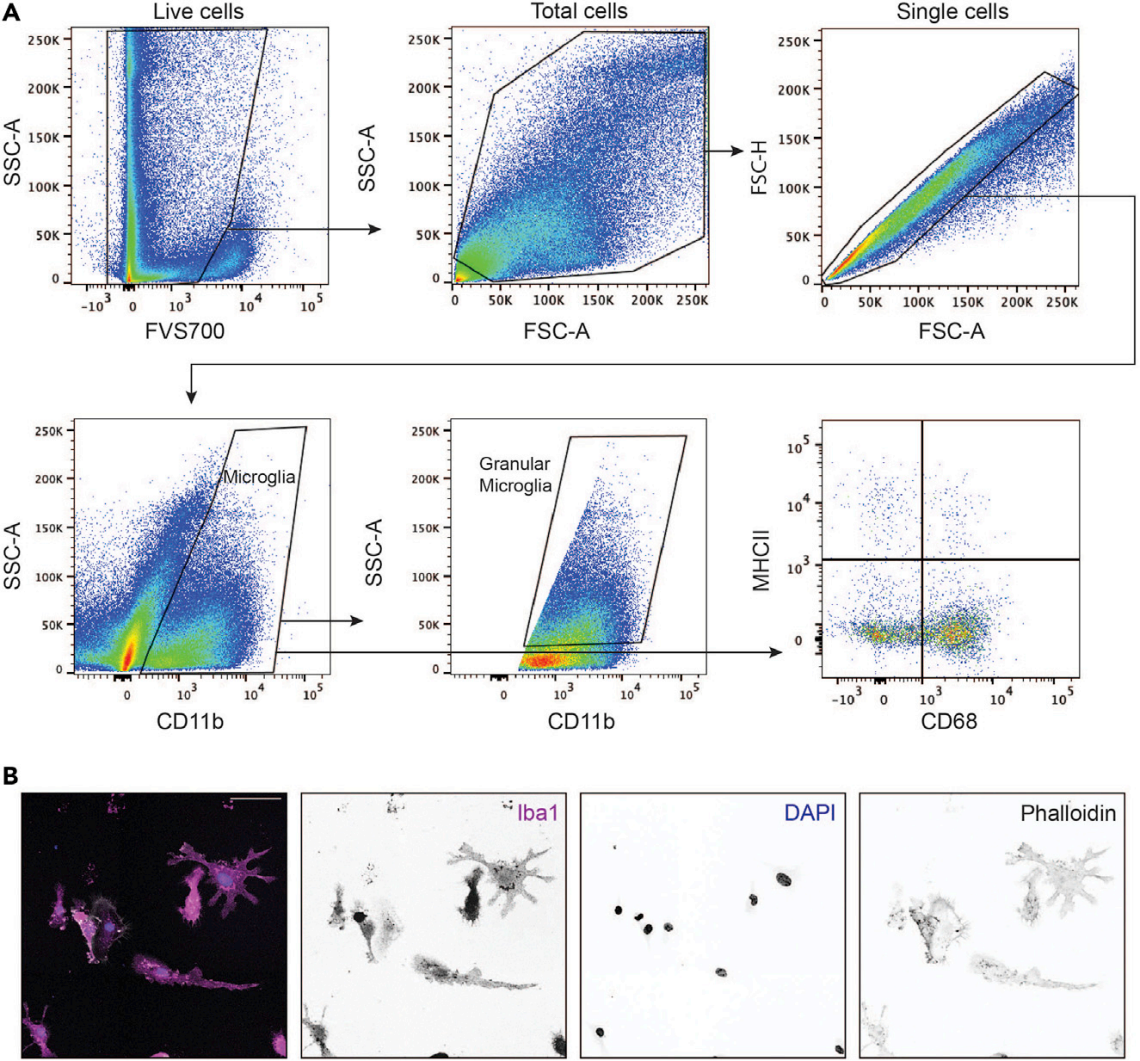
Adult glia flow cytometry gating strategy and microscopy (A) Gating strategy for CD11b^pos^ adult microglia, and markers for aged mice (CD68^pos^MHCII^pos^). (B) Immunocytochemical staining of purified adult microglia (Iba1^pos^). Scale bars, 50 μm.

4. Resuspend the bulk brain cell suspension obtained in step 2 above, using 90 μL MACS buffer per 1 × 10^7^ cells (an adult brain will usually yield ∼3 × 10^6^ cells in total).
5. Add 10 μL of anti-CD11b magnetic beads per 1 × 10^7^ cells (if cell yield is less than 1 × 10^7^, use 10 μL anti-CD11b magnetic beads as a minimum).
6. Incubate at 4°C for 15 min to sufficiently ‘label’ the microglia.
7. Add 2 mL MACS buffer to the tube to wash the cells.
8. Pellet the cells by centrifugation at 300 × *g* for 10 min at 4°C and aspirate the cell supernatant.
9. Meanwhile, prepare a MACS MS column as follows (each MS column can process ≤1 × 10^7^ cells).
  a. Place the MS column onto an OctoMACS magnetic separator.
  b. Add a 70 μm cell strainer to the top of the column and apply 500 μL MACS buffer to the strainer, so that the buffer flows through the column.
10. Resuspend the cells from step 8 in 500 μL MACS buffer and apply the suspension to the MS column through the 70 μm cell strainer.
11. Wash the cell strainer and column 3 times with 500 μL MACS buffer.
12. Add 1 mL MACS buffer to the MS column and remove it from the magnetic strip, then expel the MACS buffer through the column using the supplied plunger into a fresh 15 mL Falcon tube (this constitutes the ‘CD11b^pos^ cells’ fraction, containing microglia).
13. Pellet the CD11b^pos^ cells by centrifugation at 300 × *g* for 10 min at 4°C, and then aspirate the cell supernatant.
14. Resuspend the CD11b^pos^ cell pellet in 300 μL AGM.
15. Perform flow cytometry to determine the microglial purity at day 0 (Figures 2A and 3).

**Figure 3:**
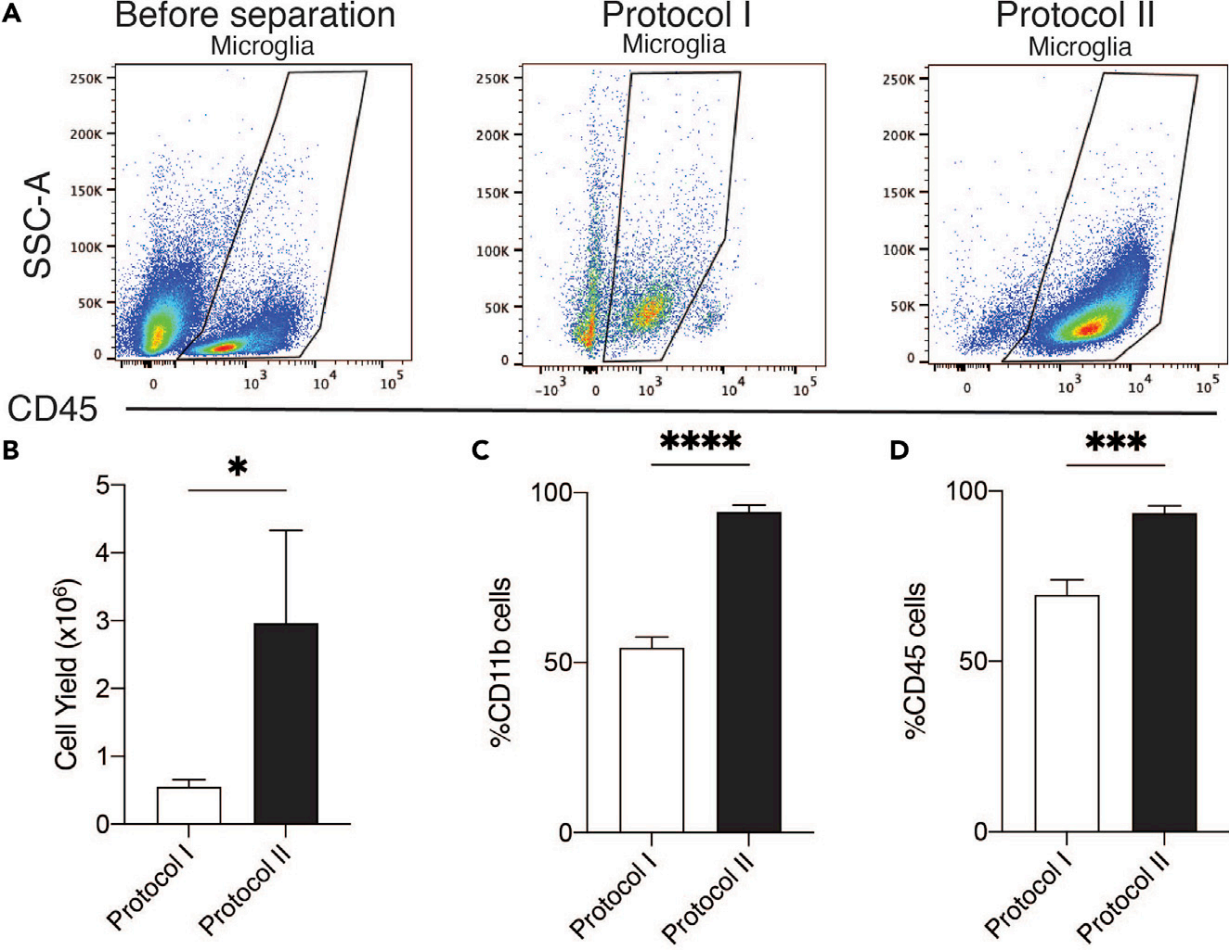
Protocol II outperforms Protocol I in both microglial yield and purity (A–D) Representative flow cytometry plots showing microglial purity using Protocol II (day 12) compared to Protocol I (day 0). Comparison of the two protocols for (B) viable cell yield per adult brain, and (C and D) percentage of CD11b^pos^ and CD45^pos^ cells in microglial preparations. Data are mean plus SEM of n=5–11 independent experiments. Data were analyzed using an unpaired student t-test (∗ p<0.05, ∗∗ p<0.01, ∗∗∗ p<0.001, ∗∗∗∗ p<0.0001).
16. Count live cells using a hemocytometer with Trypan Blue exclusion.
17. Seed microglia at 3 × 10^5^ cells per cm^2^ well size in AGM containing 50 ng/mL GM-CSF and 100 ng/mL M-CSF, and culture the cells in a 37°C/5% CO_2_ incubator for 5 days.
  a. Change the culture media the day after seeding, and every subsequent second day.
  b. Assay microglia for phenotypes of interest on day 5 after seeding.

### Protocol II: Isolation of adult microglia and astrocytes using mechanical and magnetic sorting

**Timing: 11–14 days**

This protocol allows for the successful culture and isolation of pure adult microglia and astrocytes for *in vitro* characterization. It uses mechanical separation to obtain microglia, and a CD11b magnetic sort system to negatively sort astrocytes, which do not express CD11b (https://www.miltenyibiotec.com/AU-en/products/cd11b-microbeads-human-and-mouse.html#gref).

**Figure 4:**
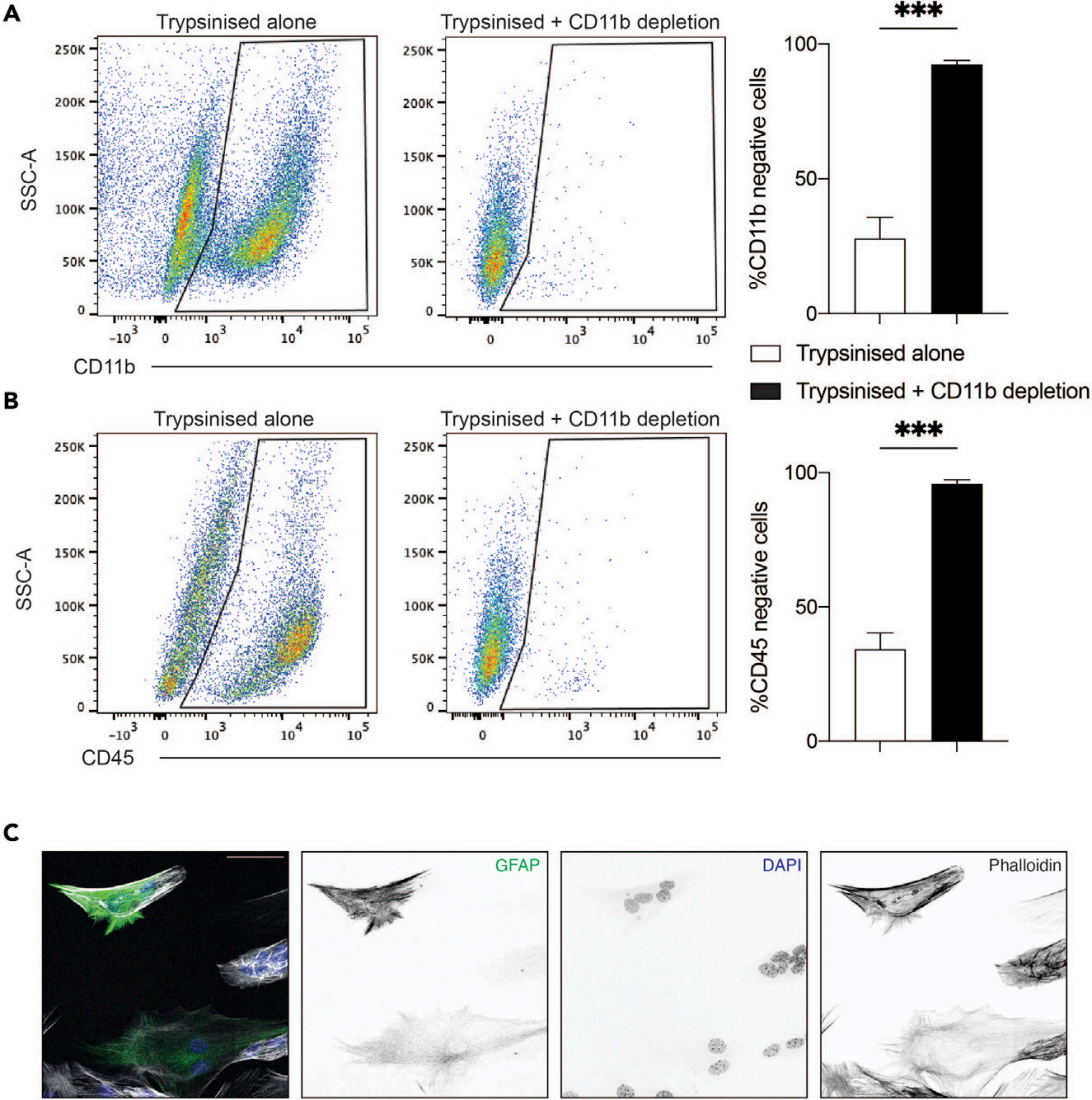
Protocol II can be utilized to purify adult astrocytes (A and B) Representative flow cytometry plots showing that CD11b depletion after trypsinization in Protocol II removes contaminating microglia (CD11b^pos^CD45^pos^) and increases the purity of astrocyte (CD11b^neg^CD45^neg^) cultures. (C) Representative immunocytochemistry of adult astrocyte cultures obtained using CD11b depletion in Protocol II. Scale bars, 50 μm. All results are from n=3–4 independent experiments. Graphs are mean + SEM, with significance determined by an unpaired student’s t test (∗ p<0.05, ∗∗ p<0.01,∗∗∗ p<0.001, ∗∗∗∗ p<0.0001).
23. Seed astrocytes in AGM at 3 × 10^5^ cells per cm^2^ well size.
  a. Change the astrocyte medium every second day after seeding.
  b. Assay astrocytes for phenotypes of interest on day 5 after seeding.

***Note:*** The protocol results in high astrocyte purity on the day of isolation, and unwanted cells do not appear in the culture over time. Due to separation via the MS column, it is recommended to leave the astrocytes for 5 days in culture to recover from the isolation protocol and adhere to the culture substrate.

18. Resuspend the bulk brain cell suspension obtained in step 2 above in 7.5 mL AGM containing 50 ng/mL GM-CSF and 100 ng/mL M-CSF. Add to a T25 cell culture grade flask (day 0).
19. Culture adult mixed glial cells in a 37°C/5% CO_2_ incubator for 11–14 days, as follows:
  a. On day 5 of culture, remove half the media and discard. Replace with fresh AGM containing 50 ng/mL GM-CSF and 100 ng/mL of M-CSF.
  b. On day 10 in culture, remove all media from the flask and replace with fresh AGM containing 50 ng/mL GM-CSF and 100 ng/mL of M-CSF.

20. Isolate pure adult microglia from mixed glial culture, as follows:
  a. On days 11–14, top up media to a total of 10 mL and shake the flask at 180 rpm at 37°C without CO_2_ for 2.5 h.
    ***Note:*** Cells are usually confluent by day 11 and suitable to harvest. However, if there is a lower cell yield, or the cells are less confluent, culturing can be extended slightly (until day 14) before harvesting.
  b. After cell shaking, transfer the culture media (containing microglia) from the flask to a 50 mL Falcon tube.
  c. Collect any remaining cells adhering to the flask with 5 mL ice-cold PBS and add this wash to the Falcon tube containing the microglia from the previous step.
  d. Centrifuge the Falcon tube containing the shaken media and wash solutions at 300 × *g* for 10 min at RT to pellet the microglial cells.
  e. Aspirate the cell supernatant and resuspend the microglial cell pellet in 300 μL AGM.
  f. Count live cells using a hemocytometer with Trypan Blue exclusion.
  g. Perform flow cytometry to determine microglial purity (Figure 2 + see section “Characterisation and analysis of adult and aged glial cells by flow cytometry”).
  h. Seed microglia at 3 × 10^5^ cells per cm^2^ well size in AGM containing 50 ng/mL GM-CSF and 100 ng/mL M-CSF.
  i. Culture the cells in a 37°C/5% CO_2_ incubator for 2 days and assay microglia for phenotypes of interest on day 2 after seeding.
    ***Note:*** Protocol II can achieve high microglial purity on the day of isolation, and unwanted cells do not appear in the culture over time. It is thus recommended to leave the microglia for 2 days in culture after the isolation protocol such that they can attach to the well and fully elongate.

21. Isolate adult astrocytes from the mixed glial culture, as follows:
  a. To obtain the astrocyte-enriched population still adhering to the flask after step 20c, add 2.5 mL of pre-warmed 37°C trypsin-EDTA to the flask and incubate in a 37°C/5% CO_2_ incubator for 5 min.
  b. Disrupt the astrocyte layer by firmly tapping the flask underside and return to the incubator for a further 5 min.
  c. Once again disrupt the astrocyte layer by firmly tapping the flask underside. Add 5 mL of AGM to the flask to inactivate the trypsin.
  d. Using a cell scraper, gently scrape any cells that remain adherent to the flask.
  e. Transfer the cell culture media from the flask to a new 50 mL Falcon tube.
  f. Pellet the cells by centrifuging at 300 × *g* for 10 min at RT and aspirate the cell supernatant.
  g. Resuspend the cell pellet in 2 mL PBS and apply to a new 50 mL Falcon tube via a 70 μm cell strainer to remove debris and obtain a single cell suspension.
  h. Wash the cell strainer with 10 mL PBS into the Falcon tube to collect any remaining cells.
  i. Centrifuge this tube at 300 × *g* for 10 min to pellet the cells and aspirate the supernatant.
  j. Resuspend the cells in 90 μL MACS buffer per flask (×1 flask should yield ∼1–2 × 10^6^ cells).
  k. Count live cells using a hemocytometer with Trypan Blue exclusion.
22. Purification of adult astrocytes using magnetic separation:
  a. Add 10 μL of anti-CD11b magnetic beads per 1 × 10^7^ cells (if the yield is less than 1 × 10^7^ cells, use a minimum of 10 μL anti-CD11b magnetic beads).
  b. Incubate at 4°C for 15 min.
  c. Add 2 mL MACS buffer to wash the cells.
  d. Pellet the cells by centrifugation at 300 × *g* for 10 min at 4°C and aspirate the cell supernatant.
  e. Meanwhile, prepare a MACS MS column (each MS column can process ≤1 × 10^7^ cells).
    i. Place the MS column onto the OctoMACS separator.
    ii. Place a 70 μm cell strainer on top of the column and apply 1 mL MACS buffer to the strainer so it equilibrates the column.
    iii. Place a 15 mL Falcon tube underneath the MS column to collect the CD11b-negative flow-through fraction (‘purified astrocytes’ tube).
  f. Resuspend the cell pellet from step 22d in 500 μL MACS buffer, and apply these cells to the MS column via the 70 μm cell strainer.
  g. Wash the cell strainer and column 3 times with 500 μL MACS buffer, and collect all flow-through fractions into the ‘purified astrocytes’ tube.
  h. Pellet the astrocytes by centrifugation at 300 × *g* for 10 min at 4°C and aspirate the cell supernatant.
  i. Resuspend the astrocyte pellet in 300 μL AGM.
  j. Count live cells using a hemocytometer with Trypan Blue exclusion.
  k. Perform flow cytometry and immunohistochemistry to determine cell purity (Figure 4).

### Characterization and analysis of adult and aged glial cells by flow cytometry

**Timing: 3 h**

**Figure 5:**
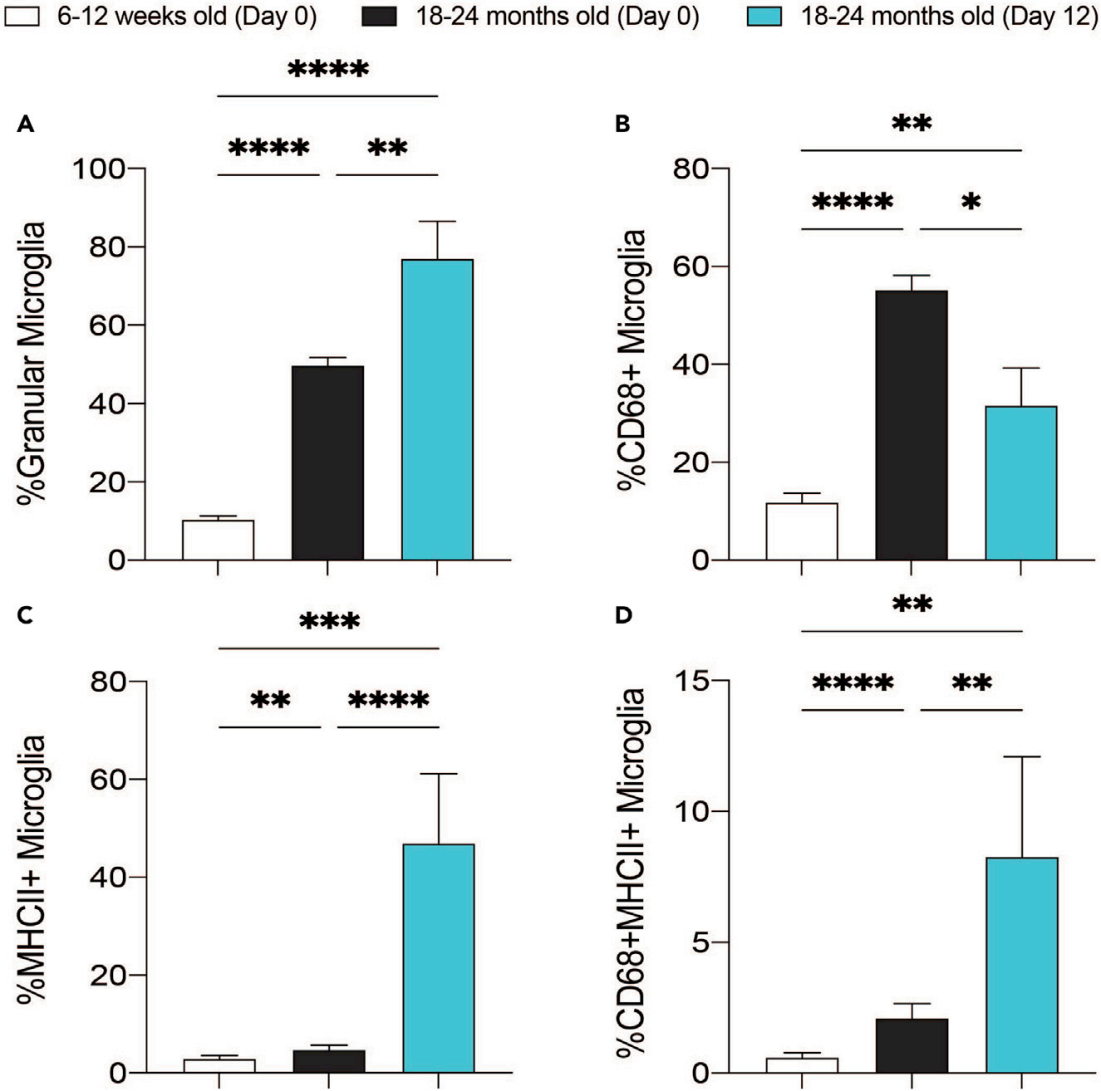
Aged microglia possess specific age-related markers that are distinguishable on day of harvest (Day 0) and maintained throughout Protocol II culturing period (Day 12) (A–D) Graphical and statistical analysis of young and aged CD11b^pos^ microglia, in terms of (A) high granularity (side scatter), (B) CD68 expression, (C) MHCII expression, and (D) CD68 and MHCII expression as measured by flow cytometry. Data are mean + SEM from n=8 independent experiments, where significance was determined by mixed effects analysis (∗ p<0.05, ∗∗ p<0.01,∗∗∗ p<0.001, ∗∗∗∗ p<0.0001).

This protocol describes a reliable way to assess microglial and astrocytic cell preparation purity using flow cytometry. CD45 and CD11b are ideal surface markers to both evaluate the microglial culture purity (CD45^pos^CD11b^pos^) and measure the astrocytic culture purity (CD45^neg^CD11b^neg^). This protocol also demonstrates how to assess cell aging markers in microglia isolated from young and aged mice by flow cytometry. Well-studied markers of aging were used to establish a baseline of our age-related phenotype (on day 0, immediately after isolation from brain), and then to assess any changes in these markers after the culturing period (day 12) (Figures 2 and 5). These markers include microglial granularity (as measured by side scatter in flow cytometry) (Ritzel et al., 2015), CD68 expression (O'Neil et al., 2018; Farso et al., 2013), and MHCII expression (Streit et al., 2020) (Figure 5).

24. Stain cells for cell surface markers.
  a. Stain cells in 50 μL PBS in a 96-well round bottomed plate for 20 min on ice in the dark (see Materials and equipment for antibody details).
  b. Prepare unstained and single stained samples to set appropriate PMT voltages on the flow cytometer and to distinguish positive and negative populations.
  c. Prepare fluorescence minus one (FMO) samples to set appropriate gates during analysis and to distinguish positive from negative populations.
  d. Wash cells by applying 150 μL PBS and centrifuging at 600 × *g* for 5 min at RT. Aspirate the cell supernatant.
  e. If not staining for intracellular markers (e.g., CD68), resuspend the cells in 100 μL PBS and transfer cells to FACS tubes already containing 150 μL PBS.
25. Stain cells for intracellular markers.
  a. Resuspend cells in 100 μL BD FixPerm solution and incubate at 4°C for 20 min in the dark.
  b. Wash cells by applying 100 μL 1× BD FixPerm Wash buffer and centrifuging at 600 × *g* for 5 min. Aspirate the cell supernatant.
  c. Stain for intracellular markers (e.g., CD68) in 50 μL 1× BD FixPerm Wash buffer (see Materials and equipment for antibody details).
  d. Incubate the cells on ice in the dark for 15 min and then wash by applying 150 μL 1× BD FixPerm Wash buffer and centrifuging at 600 × *g* for 5 min. Aspirate the cell supernatant.
  e. Aspirate the cell supernatant and resuspend the cells in 100 μL PBS.
  f. Transfer cells to FACS tubes already containing 150 μL PBS.
26. Analyze cells using flow cytometry.
  a. Run cells through a flow cytometer (e.g., BD LSR Fortessa X-20) to analyze marker expression.
  b. Use unstained and single-stained samples to set appropriate PMT voltage, and to distinguish positive and negative populations.
  c. Prepare additional single-stained samples to measure for auto-compensation.
27. Analyze flow cytometry data.
  a. Export data from the flow cytometer software (e.g., FACSDiva as FCS files), and load these into analysis software such as FlowJo.
  b. Use FMO samples to determine the gating strategies described in Figure 1 to analyze marker expression.
  c. Use a general data analysis software (e.g., GraphPad Prism) to plot percentages of interest and to assess and compare purity and phenotypes across samples and biological replicates.

### Characterization and analysis of adult glial cells by immunocytochemistry

**Timing: 3 h**

This protocol describes how to prepare adult and aged microglia and astrocytes for analysis by immunocytochemistry with cell-specific markers (Figures 2 and 4). For this purpose, adult mice are defined as 6–12 weeks old and aged mice as 18–24 months old.

28. Coat 12 mm 1.0′ coverslips with 500 μL Poly-L-lysine solution (1:10 dilution in water) by incubating for 1 h at RT in a 24-well plate. Wash coverslips three times with sterile PBS and leave overnight at 4°C to dry.
29. Plate cells on coverslips and culture cells for 2–5 days, as follows:
  a. Following shaking in step 20 above, plate adult microglia (100,000 cells/500 μL) on Poly-L-lysine-coated coverslips and culture in a 37°C/5% CO_2_ incubator for an additional 2 days.
  b. Following CD11b magnetic depletion in step 22 above, plate astrocytes (50,000 cells/500 μL) and culture in a 37°C/5% CO_2_ incubator for an additional 5 days.
30. Aspirate the cell culture media and wash cells twice with PBS.
31. Fix cells with 4% paraformaldehyde (PFA) for 15 min at RT. Wash coverslips thrice with PBS.
32. Block unspecific binding site with 0.5% BSA/PBS for 30 min at RT.
33. Stain cells with cell-specific markers (see Materials and equipment for antibody details) in 0.5% BSA/PBS for 1 h at RT. For example, use anti-Iba1 antibody (1:500 dilution, 1 μg/mL final concentration) and/or anti-GFAP antibody (1:500 dilution, 1 μg/mL final concentration).
34. Wash the coverslips 5 times (5 min each) with PBS.
35. Stain cells (see Materials and equipment for antibody details) in 0.5% BSA/PBS for 1 h at RT. For example, use DAPI (1:1,000 dilution, 20 ng/mL final concentration), phalloidin 647 (1:40 dilution), Alexa Fluor 488 anti-goat (1:500 dilution, 4 μg/mL final concentration) and Alexa Fluor 594 anti-rabbit (1:500 dilution, 4 μg/mL final concentration) in 0.5% BSA (1 h, RT).
36. Wash the coverslips 5 times (5 min each) with PBS.
37. Mount the coverslips on microscope slides with Prolong gold antifade reagent. Coat the edges of the coverslip with a thin layer of clear nail polish and leave to dry (1 h, RT). Store at 4°C for long term storage.

***Note:*** Samples were visualized on a Zeiss LSM710 AiryScan NLO with ZEN software. Image analysis was completed using the program FIJI. Background signal was subtracted from images using the Math function on the FIJI software.

### Evaluation of protocol I versus protocol II

Previous publications in the field have utilized Percoll gradients and magnetic separation of brain homogenates to isolate and culture microglia for *in vitro* characterization (Protocol I) (Mela et al., 2020; Guillot-Sestier et al., 2021; Grabert and McColl, 2018; Njie et al., 2012; Bohlen et al., 2019). However, such methods of isolation and purification result in low cell yield and low cell viability, making it difficult to perform multiple assays with the same cells (Figure 3). In comparison, our newly developed method (Protocol II) allows for the successful culture of both young and aged murine microglia and astrocytes, while optimising cell preparation workflows to obtain a high yield of pure microglia and astrocytes from adult and aged brains (Figure 2, Figure 3, Figure 4).

We have described a robust protocol to obtain pure microglia from adult and aged brains, using a method that also enables astrocyte purification using CD11b magnetic depletion. After shaking the microglia from the flask, some CD11b+ microglia remain attached to the flask, and can contaminate the astrocyte population remaining adherent to the flask. To generate pure astrocyte cultures, it is thus critical to deplete CD11b-positive cells from the astrocyte fraction by magnetic separation (Figure 4).

While Protocol II employs a longer culturing protocol than Protocol I, it also maintains the age-related phenotypes of the microglia that are observed on day 0 (the day of harvest) (Figure 5). Protocol II is thus the protocol of choice for *ex vivo* investigations of microglia aged *in vivo*. Protocol II evaluates the % microglia in total brain homogenate at day 0 (the day of harvest), while purity of microglia is only assessed at day 11 after culturing the mixed glia culture.

***Note:*** Upon using either Protocol I or II, the cultured microglia present an activated status and start to upregulate surfaces markers such as MHCII, as expected in the presence of GM-CSF. Importantly however, the Protocol II microglia maintain the age-related phenotype present at day 0.

## Expected outcomes

Protocol II generates viable and pure single-cell suspensions of both microglia and astrocytes. These cells can be further processed for analysis using a range of techniques, including flow cytometry, *in vitro* phenotyping, western blot, RT-qPCR and immunocytochemistry.

Protocol II provides a consistent and reliable method to obtain microglia and astrocytes from young and aged adult mice. This newly applied technique results in cultures with greater purity and yield of viable cells than conventional techniques (Protocol I). It will also enable new investigations of the molecular and biochemical mechanisms within these important brain cells, and facilitate future studies to understand age-related changes that occur in glial cells in health and disease.

## Limitations

Here we describe two distinct protocols for isolating microglia and astrocytes. Each protocol has its own intrinsic limitations. Performing a magnetic sort on day of harvest (Protocol I) will obtain fewer cells compared to culturing total cells in a flask (Protocol II). However, culturing the cells in a flask for 11–14 days (Protocol II) can subtly or dramatically alter cell phenotypes compared to 5 days in culture following a magnetic sort (Protocol I). In both protocols, microglia are cultured in the presence of M-CSF and GM-CSF, which both promote cell survival and proliferation. However, these growth factors can modulate expression levels of age-related cell markers, as seen most dramatically in Figure 5C, where MHCII expression is significantly higher after 12 days in culture compared to day 0. In keeping with this, GM-CSF appears to modulate MHCII expression in human monocytes (Hornell et al., 2003). Despite the changes in these select age-related markers, we still observed a sustained upregulation of CD68 and MHCII markers over the culturing period in microglia isolated from aged mice, suggesting that these cells are, to an extent, able to retain some of their age-related phenotype. Ideally, assessment of additional age-related and senescent markers, such as β-galactosidase activity, HMGB1 release, and IL-6 secretion, could be measured to further confirm the microglial phenotype after 12 days in culture. Overall, Protocol II shows an increased yield and purity, which alongside its partially age-maintained phenotype, makes it a superior method to isolate adult and aged primary microglial cells.

Another limitation of both Protocol I and II is the requirement for a Miltenyi Biotec OctoDissociator with heaters, and the Miltenyi Biotec OctoMACS separator. This equipment is essential to maximize total brain cell yield and effective magnetic separation of the mixed glia. If the Miltneyi Biotec OctoDissociator with heaters is unavailable, we recommend to instead digest the adult brain with the Miltenyi Biotec Adult Brain Dissociation Kit in a 37°C shaking incubator (see below, “Troubleshooting”). However, some form of magnetic sort must be performed to remove contaminating microglia from the astrocytes preparation if pure cultures are required. Additionally, this protocol utilizes the entire adult brain including the cerebellum, and we have not tested this protocol for microglial isolation from specific brain regions. For this to be attempted, we would recommend pooling specific brain regions from several mice in order to obtain enough cells to form confluent layers in a T25 flask.

Protocol II also enables astrocytes to be obtained simultaneously, making it an attractive isolation protocol compared to Protocol I. However, assessing and quantifying pure astrocyte populations remains a limitation of Protocol II. In addition to the CD45^neg^CD11b^neg^ population (Figure 4), GLAST is often used as an astrocyte marker on flow cytometry, but presents a challenge. Astrocytes express GLAST heterogeneously, making it difficult to define a true positive and negative population due to a continuum of expression. Additionally, astrocyte surface markers tend to be downregulated in suspension and thus are difficult to detect via flow cytometry. We found measuring astrocytic GFAP expression by immunocytochemistry to be a more robust readout of astrocyte purity, particularly when the cells have had multiple days to rest in culture. At the same time, immunocytochemistry enables the assessment of cell morphology, and observation of additional distinct phenotypes, confirming astrocyte purity and excluding any microglia contamination.

## Troubleshooting

### Problem 1

Do not have Miltneyi Biotec gentleMACS OctoDissociator with heaters for brain homogenization (step 2d).

### Potential solution

Instead use a Miltenyi Biotec Adult Brain Dissociation Kit and a 37°C shaking incubator at 180 rpm. Resuspend and gently homogenize the brain every 5 min for a total of 30 min. This alternative method generates a lower yield of viable cells (30%–40% reduction) due to increased cell damage.

### Problem 2

Debris removal step does not sufficiently clear cell debris (step 2o).

### Potential solution

Ensure that the volume of brain homogenate does not exceed 3,100 μL before applying the debris removal solution. Brain homogenate volumes exceeding 3,100 μL will over-dilute the debris removal solution. This will result in insufficient clearance of brain tissue and leaves an undefined layer of debris that is difficult to remove. If the debris removal is insufficient, remove what debris can be separated, and continue with the protocol. For Protocol I, the CD11b magnetic selection should remove most of the debris, however the cells may take a few more days to recover (6–7 days). For Protocol II, it is recommended to completely change the flask media after 2 days in culture, gently wash the cells with warm media, and then add fresh AGM containing 50 ng/mL GM-CSF and 100 ng/mL M-CSF.

### Problem 3

The adult mixed glial culture in the flask is not 80% confluent on day 11 (step 20; see Figure 1E for example of confluent vs. non-confluent cultures).

### Potential solution

There are several potential solutions to this problem:

• Change the media fully and leave the flasks to culture for 2–3 more days (do not exceed 14 days in culture). This should promote proliferation of the astrocytes underneath the microglia, allowing the microglial layer to be successfully shaken off.
• If the cells do not proliferate further over this extra culture period, skip the mechanical (shaking) separation step of the protocol (step 20a) and proceed directly to step 21. Shaking a non-confluent flask does not yield many cells, and instead liberates unwanted cell debris.
• If the problem persists, consider adding ×1.5–2 adult brains to a single T25 flask, to increase the number of cells in the flask and thereby increase cell confluency.

### Problem 4

Microglia contaminate the purified astrocyte fraction, even after magnetic CD11b depletion (step 22h).

### Potential solution

• Microglia can sometimes escape removal by CD11b sorting and contaminate the flow through (which should be CD11b-negative). One solution is to use LS columns instead of MS columns; this will increase the opportunity for microglia to stick to column to improve CD11b depletion efficiency in the flow-through fraction.
• It is also possible to re-plate the astrocytes into a T25 flask after CD11b-negative depletion, and culture these cells until they are confluent (takes an additional 7 days in culture). Once confluent, a second round of trypsinization and CD11b depletion can be performed (steps 21+22), potentially removing the remaining microglial contamination.

### Problem 5

The adult astrocyte culture displays heterogeneous expression of GFAP (from intensely bright cells to a low signal) (step 33).

### Potential solution

• GFAP is heterogeneously expressed in astrocytes both *in vitro* and *in vivo.* A potential solution would be to co-stain with another astrocyte-specific marker, such as S100β.
• Factors such as astrocyte culture density, health, morphology, and age affect the GFAP expression and signal by immunocytochemistry. A potential solution is to plate astrocytes at a higher cell density to improve culture health and therefore the level of GFAP expression.
• Plating astrocytes on a 2D surface such as a glass coverslip also impedes general cell viability, which is suboptimal for immunocytochemistry. A potential solution is to culture astrocytes in a 3D environment such as Matrigel-coated coverslips. This may improve astrocyte morphology and health, and in turn improve GFAP signals for immunocytochemistry (Balasubramanian et al., 2016).

## Resource availability

### Lead contact

Further information and requests for resources and reagents should be directed to and will be fulfilled by the lead contact, Kate Schroder (k.schroder@imb.uq.edu.au).

### Materials availability

This study did not generate new unique reagents.

## Data Availability

This study did not generate/analyze datasets/code.
